# Concomitant surgical ablation for atrial fibrillation (AF) in patients with significant atrial dilation >55 mm. Worth the effort?

**DOI:** 10.1186/s13019-015-0337-3

**Published:** 2015-11-14

**Authors:** Simon Pecha, Samer Hakmi, Irina Subbotina, Stephan Willems, Hermann Reichenspurner, Florian Mathias Wagner

**Affiliations:** 1 Department of Cardiovascular Surgery, University Heart Center Hamburg, Martinistr 52, 20246 Hamburg, Germany; 2 Department of Cardiology, Electrophysiology, University Heart Center Hamburg, Hamburg, Germany

**Keywords:** Surgical ablation, Atrial fibrillation, Enlarged left atrium

## Abstract

**Background:**

Concomitant Surgical AF ablation is an established procedure, recommended in guidelines. However many surgeons are reluctant to perform AF ablation in patients with significantly enlarged left atrium. We therefore analyzed outcomes of patients with left-atrial diameter >55 mm undergoing concomitant AF ablation.

**Methods:**

Between 05/2003 and 12/2012 124 patients with significantly enlarged left-atrium >55 mm underwent concomitant surgical AF ablation. Rhythm monitoring was accomplished by implantable loop recorder (ILR) interrogation (*n* = 54), or 24-h Holter-ECG (*n* = 70). Successful ablation was defined as AF Burden <0.5 % in ILR interrogation or absence of AF episode >30 s in 24-h Holter-ECG. Primary endpoint of the study was freedom from AF at 12 months follow-up.

**Results:**

Mean patient’s age was 65.7+/−9.6 years, 69.4 % were male. No major ablation or ILR related complications occurred. Mean LA diameter was 60.7+/−4.4 mm. Survival rate at one-year follow up was 94.4 %. 11 (8.8 %) patients received additional catheter-based ablation, while 23 (18.5 %) had an electrical cardioversion during follow-up period. Overall freedom from AF rate after one-year follow-up was 64.4 % and 59.4 % off antiarrhythmic drugs respectively. Logistic regression analysis identified preoperative paroxysmal AF, duration of AF and LA diameter > 70 mm as predictors for rhythm outcome at 12 months follow-up.

**Conclusion:**

In this patient cohort with significantly enlarged LA diameter, concomitant surgical AF ablation provided freedom from AF of 64.4 % after one-year follow-up. However in this patient population, an accurate postoperative care with interventions like medical or- electrical cardioversion and additional catheter based ablation is necessary to achieve satisfactory results.

## Background

Atrial fibrillation (AF), the most common arrhythmia in patients undergoing cardiac surgery, is associated with an increased risk of death, stroke, and hospitalisation, and is furthermore known to reduce quality of life and exercise capacity [1, 2]. Therefore, the 2012 Guidelines for the management of AF issued by the European Society of Cardiology (ESC), European Association of Cardiothoracic Surgery (EACTS), and European Heart Rhythm Association (EHRA) recommend concomitant surgical AF ablation for symptomatic patients, as well as asymptomatic patients with low risk for an ablation procedure [3]. Cox first reported his technique of surgical AF ablation using the cut-and-sew principle in 1987. This technique was modified and resulted in the so-called Cox maze III procedure, which, because of its excellent results, with success rates > 90 %, remained the gold-standard for surgical AF ablation for many years. However, owing to its complexity, only a few surgeons performed this procedure. Within years, the cut-and-sew principle was replaced by the application of various energy sources to create transmural atrial lesions, and the use of the procedure became widespread [4]. In recent studies published success rates of surgical AF ablation are ranging between 65 % and 90 %, depending on preoperative type of AF, used lesion set and follow-up method [5–10]. Some studies, investigating predictors for rhythm results after AF ablation, have shown, that an enlarged left-atrium >55 mm is associated with a higher risk of ablation failure [10–13]. Therefore, many surgeons are reluctant to perform concomitant surgical ablation procedures in this patient cohort. However there is only little relevant information in the literature, and no clear data exist if there is any certain LA diameter which is associated with impaired results and should probably be used as a cut-off value. Therefore, we aimed to investigate the safety and efficacy of concomitant surgical AF ablation in patients with a severely enlarged LA diameter >55 mm.

## Methods

Between May 2003 and December 2012, 603 patients underwent concomitant surgical ablation in our institution. In 124 of these patients a severely enlarged left atrium (>55 mm) was observed on preoperative echocardiography. 82 (66.1 %) patients were treated due to persistent or longstanding-persistent AF, while 42 (33.9 %) received ablation for paroxysmal AF. Data of these patients were prospectively collected into a database and a retrospectively analyzed.

Isolated bilateral pulmonary vein ablation was performed in 12 (9.7 %) patients. Complete left atrial ablation including pulmonary vein isolation, box lesions, and left atrial appendage and isthmus isolation was performed in 78 (62.9 %) patients. Biatrial ablation was conducted in 34 (27.4 %) cases, which included additional right atrial intercaval lesion, isolation of the cavotricuspid isthmus, right atrial appendage and terminal crest.

Pulmonary vein isolation was only used in patients with paroxysmal AF, while biatrial ablation was only used in patients with persistent AF. Complete left-atrial lesion set was used in 30 patients with paroxysmal AF and in 48 patients with persistent- or longstanding persistent AF. Relatively low number of patients receiving biatrial lesion set is associated to the fact that we first started to use biatrial ablation in our institution in 2008. Nowadays, patients with longstanding-persistent AF all receive a biatrial lesion set.

The energy sources applied included argon-based cryoablation (cryoICE cryo-ablation probe, AtriCure Inc., West Chester, OH, USA; Cardioblate CryoFlex Surgical Ablation Probe, Medtronic Inc., Minneapolis, MN, USA) in 24 patients, unipolar radiofrequency ablation (Cardioblate unipolar RF pen, Medtronic Inc.) in 51 patients, and bipolar ablation (Cardioblate BP2 device and Cardioblate Surgical Ablation System Generator, Medtronic Inc.) in 39 patients.

### Statistical analysis

All statistical analyses were performed using SPSS statistical software version 21.0 (SPSS Inc., Chicago, IL, USA). Continuous values are expressed as mean ± standard deviation and were compared using Student’s *t*-test or the Mann–Whitney test, as appropriate. Categorical variables are displayed as frequencies, and percentages were compared using the chi-square test or Fisher’s exact test, as appropriate. *P* < 0.05 was considered statistically significant. Reported *P* values are two-sided. Cox regression analysis was used to determine survival rates and predictors for freedom from AF at 1-year follow-up. ROC analysis was used to identify a cut-off value for LA diameter.

### Follow-up

All rhythm results were obtained by either implantable loop recorder (ILR) interrogation (*n* = 54), or 24-h Holter-ECG (*n* = 70) at three and 12 months’ follow-up. AF recurrence was defined as an AF burden > 0.5 % in ER interrogation or a single AF episode with duration > 30 s on the 24-h Holter ECG. Postoperative as well as discharge rhythm results were obtained using 12-lead ECG. The antiarrhythmic drugs and anticoagulation regimens were maintained for 3 months postoperatively in all patients and then adapted according to the rhythm results. In patients without contraindications, amiodarone was used as first-line antiarrhythmic drug therapy; otherwise, other class I or III antiarrhythmic drugs were used for at least three months postoperatively. Echocardiography was performed prior to the surgical procedure.

## Results

### Demographic and intraoperative data

Baseline patient characteristics are displayed in Table 1. Mean patients’ age was 68.7 ± 9.7 years. Mean left atrial diameter was 60.7+/−4.4 mm. Mean AF duration was 4.1 ± 3.8 years. Mean preoperative LVEF was 51.0 % ± 11.7 %. 8 (6.4 %) patients had a history of stroke. Performed surgical procedures were isolated coronary artery bypass grafting (CABG) in 24 patients, aortic valve replacement in 20 patients, and mitral valve repair or replacement in 22 patients. A combined CABG and valve operation was performed in 43 patients; other surgical procedures made up the remaining 15 cases. No major ablation-related complications occurred in any of the patients. 2 patients (1.6 %) experienced perioperative stroke, while 3 patients experienced stroke during 12 months follow-up period. Postoperative new permanent pacemaker implantation rate was 10.1 %.

**Table 1:** Patients’ characteristics

	Patients *n* = 124
Age (years)	68.7 ± 9.7
Gender (female/male)	38/86
AF duration (years)	4.1 ± 3.8
Paroxysmal AF n (%)	42 (33.9)
LA diameter (mm)	60.7 ± 4.4
LVEF (%)	51.0 ± 11.7
Prior Stroke n (%)	8 (6.4)
Diabetes n (%)	25 (20.2)
Renal insuffiency n (%)	15 (12.1)
COPD n (%)	10 (8.1)
Coronary artery disease n (%)	45 (36.3)
Previous MI n (%)	18 (14.5)

### Rhythm results

**Fig. 1:**
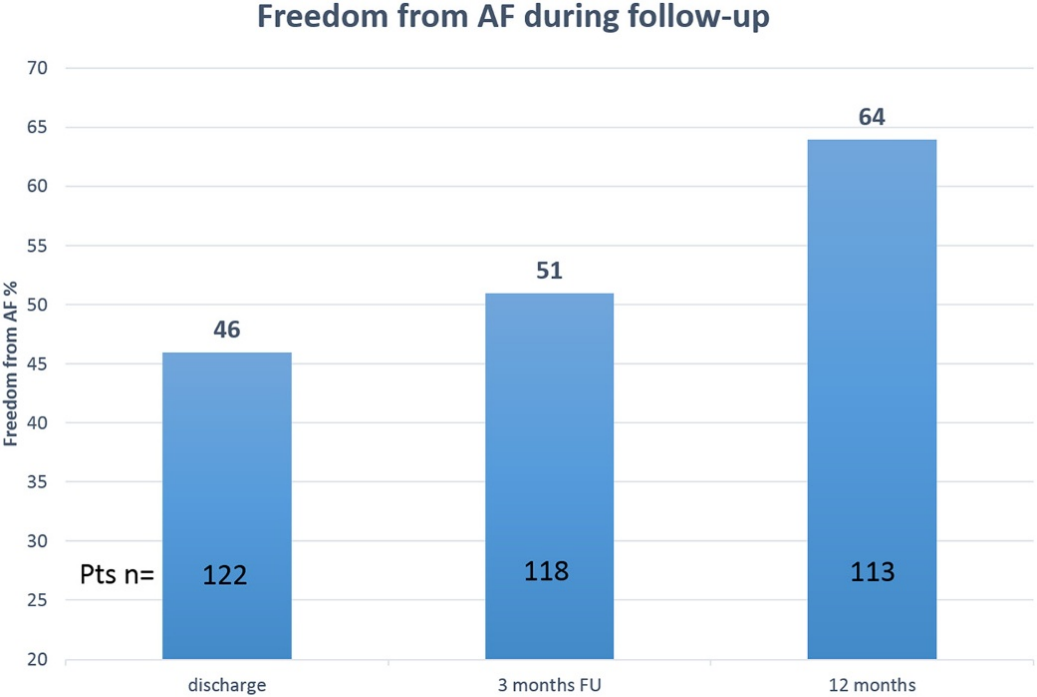
Rhythm results during follow-up

Freedom from AF was recorded by 12-lead ECG immediately after the procedure and at the time of discharge in 52 % and 46 % of patients, respectively. All patients underwent either ER interrogation (*n* = 54) or 24-h Holter ECG monitoring (*n* = 70) at three and 12 months’ follow-up. 67 patients (54.0 %) also attended a six-month follow-up. At three and six months’ follow-up, 51 % and 55 % of patients, showed freedom from AF. At 12 months’ follow-up, freedom from AF was 64.4 %, and 59.4 % off anti-arrhythmic drugs respectively (Fig. 1). Patients with preoperative paroxysmal AF had statistically significant higher rate of freedom from AF, compared to patients with persistent AF, (74.2 % vs. 55.3 %; p = 0.02).

Cox regression analysis was used to identify predictors for freedom from AF after 12 months. Preoperative paroxysmal AF (*p* = 0.017 OR: 1.36 CI: 1.08-2.31) as well as shorter duration of AF (*p* = 0.03 OR 1.14 CI: 0.98-1.70) were statistically significant predictors for freedom from AF after 12 months. Furthermore an LA diameter >70 mm was identified in ROC analysis to be a cut off value, predicting ablation failure. Neither demographic data, indication for surgery, lesion set and used energy source had an impact on SR rate after one year.

### Additional procedures

In patients with persistent AF at the time of follow up, electrical cardioversion was initiated. Patients with recurrence of AF 6 months postoperatively were considered for additional catheter-based ablation, if reasonable. During follow-up period, 23 (18.5 %) received electrical cardioversion, while 11 (8.8 %) had an additional catheter based-ablation. In patients receiving catheter ablation, electrophysiological mapping was performed. 5 patients had gaps in the surgically performed lines, while in 6 patients additional lines were performed. 3 of them were treated due to atrial flutter.

### Survival

There were no cases of intraoperative death. The in-hospital mortality rate was 1.8 %, while the 30-day mortality rate was 2.7 %. The one-year survival rate was 94 % for the entire patient population. There were no differences in survival between patients with successful ablation and those with ablation failure during 1-year follow-up period.

## Discussion

This study investigates the impact of concomitant surgical AF ablation in a patient cohort with significantly enlarged LA > 55 mm. We have shown, that even in this group of patients, that is considered as a population with high risk for ablation failure by many surgeons, satisfactory results for SR restoration can be achieved. Freedom from AF was 64.4 % and 59.4 % off antiarrhythmic drugs after one-year follow up in our study. We observed significantly higher SR restoration rate results in patients with preoperative paroxysmal AF, compared to those with persistent- or long-standing persistent AF, a finding which is well-known and in line with several previously published papers. Searching for predictors of ablation success, in our study, preoperative paroxysmal AF was the only statistically significant predictor in univariate logistic regression analysis. In contrast to other previously published studies, we did not observe an influence of the used lesion set on ablation success [14, 15]. Especially in patients with persistent- or longstanding persistent AF several studies have shown superior results for biatrial ablation [9, 15]. However our results may be explained due to the relatively small number of patients that received biatrial lesion set in our cohort, so that the statistical power might be too small and superior results in the biatrial group might not reach statistical significance. Furthermore we did not find any influence of the used energy source on ablation success, a result that has been also published by other groups.

Also the type of surgical procedure did not influence the rhythm outcome in patients with significantly enlarged LA. We found that patients with larger LA had more often Mitral valve disease, an observation, previously described in other studies [16].

Several studies have shown an impact of LA size on ablation success. Damiano et al. have reported that an enlarged LA diameter is a predictor for late failure after Cox-Maze IV procedure [11]. Similar observation has been previously published by our group in a cohort of 503 patients receiving surgical AF ablation. In a multivariate logistic regression analysis we also found LA size to be a predictor for ablation failure [10]. Ad et al. have published a study of patients with surgical AF ablation, where they compared outcomes of patients with LA diameter > 55 mm with those with LA diameter <55 mm. In the one-year analysis patients with small LA had superior results, but at 2 year follow-up patients with large and small atria had similar SR rates.

In our study we found that patients with enlarged LA >55 mm still have good SR rate after 1-year follow-up. However in logistic regression analysis, LA diameter of 70 mm seems to be a cut-off value where ablation results tend to be significantly worse. This finding is in line with the data published by Ad et al., they observed an LA diameter between 70 and 80 mm as predictor for failure of ablation [16].

In our patient population, surgical AF ablation was safe and feasible in this group of patients with significantly enlarged LA. We had no ablation-related mortality or morbidity in any of our patients. These findings are consistent with a previously published study of Ad et al. They showed, in a propensity-score matched analysis of 178 high-risk patients with an additive Euroscore > 6, that even in a high risk cohort, there is no additional operative risk when performing AF ablation compared to a non-ablation group [17].

The one-year survival rate was 94.4 % in our cohort without differences in patients with SR restoration and those with failure of ablation. There is only little published data on the effect of SR restoration after AF ablation on survival. According to our data, survival rates did not differ significantly between patients with- or without successful AF ablation. This finding is in contrast with a previous published study by Louagie et al., in which a successful Cox maze procedure with restoration of sinus rhythm resulted in a higher survival rate in those patients [18]. Furthermore previous published study by McCarthy et al. has shown a mid-term survival benefit for patients with AF receiving concomitant surgical ablation. In their propensity-score matched analysis including 4947 patients, they compared patients with AF receiving ablation (*n* = 423), patients with AF receiving no ablation (*n* = 129) and patients without AF. They found out that patients in the AF ablation group had lower mortality during mid- term follow-up, compared with patients with AF without ablation. The mortality rate of treated patients at last follow-up was similar to those of patients without AF [19].

The absence of a demonstrable survival benefit for patients who had sinus rhythm restoration in our study could be due to the relatively small number of patients and the limited follow-up time of 12 months.

In our patient population, 64.4 % of patients were in sinus rhythm after a one-year follow-up. All rhythm results were obtained using 24-h Holter ECG or ER monitoring, according to the 2012 published ESC/EHRA/EACTS guidelines [3]. This may be one of the reasons for the lower sinus rhythm rates compared to previous published studies where the rhythm follow-up used only 12-lead ECG [12, 20]. Another reason may be the fact that we were dealing with a population with a significantly enlarged AF > 55 mm where the success rates of AF ablation tend to be lower. However, given the potential long-term benefits of the restoration of sinus rhythm, particularly in patients concomitant surgical ablation is worth considering even in this surgically high-risk population, despite of potentially longer operative times.

Major limitation of the study is the retrospective single centre study design with the potential risk of bias by unknown confounders. Especially the inhomogeneous patient collective in our study makes it difficult to draw conclusions regarding lesion sets or energy sources in patients with significantly enlarged LA diameter. Furthermore our patient population consisted of a relatively small number of patients, and larger prospective randomized trials with more homogeneous patient population will be necessary to confirm the obtained results.

## Conclusion

In this patient cohort with significantly enlarged LA diameter, concomitant surgical AF ablation provided still a rate of freedom from AF of 64.4 % after one-year follow-up. Univariate logistic regression analysis identified LA duration, preoperative paroxysmal AF and LA diameter >70 mm as statistically significant predictors for rhythm outcome. However in this patient population, an accurate postoperative care with interventions like medical or- electrical cardioversion and additional catheter based ablation is necessary to achieve satisfactory results.
